# Five-year outcomes of western mental health training for Traditional Chinese Medicine practitioners

**DOI:** 10.1186/s12888-016-1080-6

**Published:** 2016-10-26

**Authors:** Tai Pong Lam, Ki Yan Mak, Kwok Fai Lam, Hoi Yan Chan, Kai Sing Sun

**Affiliations:** 1 Department of Family Medicine and Primary Care, The University of Hong Kong, 3/F, Ap Lei Chau Clinic, 161 Main Street, Ap Lei Chau, Hong Kong; 2 Mental Health Association of Hong Kong, Kwun Tong, Hong Kong; 3 Department of Statistics and Actuarial Science, The University of Hong Kong, Pok Fu Lam, Hong Kong

**Keywords:** Mental health, Postgraduate training, Psychiatrists, Traditional Chinese Medicine practitioners, Western medicine

## Abstract

**Background:**

There are increasing expectations for primary care practitioners to deal with mental health problems. In Hong Kong, 15 % of the general public consult Traditional Chinese Medicine (TCM) practitioners regularly for their primary health care needs. This study investigated the 5-year outcomes of a western mental health training course for TCM practitioners in Hong Kong.

**Method:**

Structured questionnaire surveys were conducted to compare the TCM practitioners’ confidence and engagement in mental health care before and after the Course. The data collected during 2011–2015 were analyzed.

**Results:**

A total of 151 TCM practitioners returned both pre- and post-Course questionnaires, with a response rate of 95.6 %. After the course, there were significant increases in the proportions of participants being confident of recognizing patients with psychological problems (62.9 % before the course vs 89.4 % after), diagnosing common mental health problems (47.7 % vs 77.5 %), and managing them (31.2 % vs 64.3 %). Overall, 66.9 % of the participants reported some increase in their confidence in recognizing patients with psychological problems, diagnosing or/and managing patients with common mental health problems. Qualitative responses illustrated the major improvements were increased awareness of mental symptoms, better understanding of classification of mental disorders and management approaches. On the other hand, barriers included difficulties in understanding medical terms in English, consultation time constraints, and a lack of formal referral system to psychiatrists.

**Conclusions:**

The Course has positive impact on TCM practitioners in handling mental health patients. The findings are useful for designing similar trainings on complementary and alternative medicine practitioners in other countries.

**Electronic supplementary material:**

The online version of this article (doi:10.1186/s12888-016-1080-6) contains supplementary material, which is available to authorized users.

## Background

Since the last decade, the World Health Organization (WHO) has promoted the integration of mental health into primary care in response to the high prevalence of common mental disorders such as anxiety and depression [1, 2]. Although there are rising expectations of primary care practitioners dealing with patients’ mental health problems, many of them lack confidence in diagnosing and managing these cases [3, 4]. To improve their competence, some postgraduate training courses have been organized for western medicine primary care doctors and significant impacts were found in different countries [5–8]. The trained doctors recognized more mental health patients compared with those without training [9–11].

In Hong Kong, besides western medicine doctors, 15 % of the general public consult Traditional Chinese Medicine (TCM) practitioners regularly for their primary health care needs [12, 13]. TCM has been widely recognized as part of the health system in Hong Kong, with strong support from the post-colonial Government since 1997 such as the launch of licensing examination and full time undergraduate programmes in universities [14]. Notwithstanding the rise of stature, TCM practitioners inclined to gain better understanding of western medicine including treatment options and evidence-based practice [14, 15]. Similar observations were shown among western studies, in which complementary and alternative medicine (CAM) practitioners addressed the need to acquire the skills of western conventional approach and guidelines [16, 17]. Studies have demonstrated the benefits of overcoming the gap between TCM and western medicine towards treating organic disease [18]. By gaining better understanding in the diagnostic and management style from western medicine doctors, the TCM practitioners showed improved interrater reliability of diagnoses. Also, better communication and agreement might facilitate future referral and collaboration.

TCM does not differentiate between psychological and physiological functions, which are seen as interconnected [19]. TCM recognizes psychosomatic problems, similar to any other organic diseases, are resulted from the disequilibrium of *yin-yang* and the disorder of *qi* and their viscera. The terms *yin* and *yang* represent two opposite yet interdependent properties of the body, whereas *qi* is the vital energy that circulates along the meridian channels to supply to all major organs. Irregular emotional activity is believed to occur as a consequence of obtruded homeostasis between the polarities, or interfered flow of energy, in the organs [20]. There is no specific classification of mental disorders in TCM. [21, 22]. Instead, they are described as symptoms in a few general terms for example depressive mood, manic mood and madness. Our published paper reported the impact of an innovative 10-session western mental health training course for TCM practitioners [23]. To our knowledge, there had been no similar study in the literature. Adopting an inter-disciplinary and problem-oriented approach, psychiatrists, clinical psychologists and family physicians were recruited to teach the training programme, in which 10 interactive seminars on topics related to common psychological problems and psychotherapy were offered (see Additional file 1). The Course aimed to increase TCM practitioners’ capacity in recognition, diagnosis, basic management and referrals of mental health patients in primary care settings. Questionnaire survey on the first batch of TCM practitioners taking the Course in 2010 reported significant improvements in their confidence in recognizing patients with psychological problems (60.6 % being confident before the Course vs. 81.8 % after), diagnosis (33 % vs. 76 %) and management (24 % vs. 55 %) of common mental health problems (e.g. anxiety, depression, sleeping disorder). Focus group interviews found that the training on western classification of mental illness was recognized as the most important reason for their improvement. We continued the pre- and post- course assessment survey subsequently for 5 years. This paper analyzed the data collected from the additional cohorts (2011–2015) which had five times the size of the previous sample (2010). Apart from a stronger quantification of their improvement in mental health care, we are also able to explore the association between their learning outcomes and background characteristics. Further, the enablers and barriers they encountered can be summarized and analyzed. The findings should be useful to other countries where TCM practitioners and other CAM practitioners make significant contributions towards their health care services.

## Methods

### Study design and data collection

Structured questionnaires were designed for the immediate pre-course and post-course based on the review of relevant literature and comments from research team members. The questionnaires were pilot-tested in December 2009 and finalized in the following month. Same set of questions was used to compare the TCM practitioners’ confidence and engagement in mental health care before and after attending the course. Open ended questions were included in the post-course survey to ask for qualitative responses about the impact of the course, barriers encountered and suggestions. The relevant questions for this study are shown in an additional file (see Additional file 2). Ethics approval was obtained from the Institutional Review Board of The University of Hong Kong/Hospital Authority Hong Kong West Cluster.

Over the five courses organized between 2011 and 2015, the students were invited to complete the pre-course survey at their first session and the immediate post-course survey after their final session. Verbal consent was obtained from the participating students when the questionnaires were handed out to them by the research assistants. In order to identify the respondent for comparisons between the anonymous pre-post- responses, each questionnaire was coded with a reference number which was known to one research assistant only and not available to other members of the research team.

### Data analysis

Quantitative analysis was carried out using the statistical software SPSS version 23.0. The measurements were mainly shown in ordinal scale, statistical inference via the nonparametric Wilcoxon signed rank test was used to determine if there were significant changes in the median of the differences in the responses before and after taking the Course. A negative value in the median of the differences, hence a negative value of the test statistics, was an indication that the participants tended more on the agreed side or a higher number reported after taking the Course. We do not have hypotheses about the direction of the changes as a consequence of taking the Course as we do not wish to impose any subjective feelings that might bias the results. Two-sided tests were used throughout this study whenever appropriate. Furthermore, Fisher’s exact test was used to test for the association between participants’ background characteristics and reported learning outcomes. A *p*-value < 0.05 was considered statistically significant.

The qualitative responses of open ended questions were analyzed with a thematic approach and grouped into common themes independently by two investigators who were both experienced in qualitative research. The consistency between the two entries was checked.

## Results

### Survey participants

The pre-course questionnaire survey had a 100 % response rate, with 158 questionnaires completed, whereas the post-course survey attained a 95.6 % response rate with a total of 151 returned valid questionnaires. Responses from the 151 participants who completed both rounds of questionnaires were used in the analysis to compare if there were significant differences between the pre- and post-survey of individual participants. Among the participants, 42.4 % were male and 57.6 % were female, all working in the public health care sector. The mean (SD) years after graduation from TCM training was 2.5 (1.71).

### Effect on confidence and engagement in mental health care

The pre- and post-course survey responses are compared in Table 1. There were significant increases in the proportions of participants being confident of recognizing patients with psychological problems (62.9 % before vs 89.4 % after), diagnosing common mental health problems (47.7 % vs 77.5 %), and managing them (31.2 % vs 64.3 %). There was also a 7.2 % increase in the proportion of participants reporting positive experiences regarding patients with mental disorders.

**Table 1:** Changes in confidence and engagement in the care of patients with mental health problems

	Pre-course (*n* = 151)		Post-course (*n* = 151)		Wilcoxon signed rank test (pre *vs* post)
	N	(%)	N	(%)	
I am confident of recognizing patients with psychological problems					
Strongly disagree	1	(0.7)	2	(1.3)	Z = −5.410, *p* < 0.001**
Disagree	55	(36.4)	14	(9.3)	
Agree	93	(61.6)	128	(84.8)	
Strongly agree	2	(1.3)	7	(4.6)	
I am confident of diagnosing patients with common mental health problems (e.g. anxiety, depression, sleeping disorder)					
Strongly disagree	6	(4.0)	1	(0.7)	Z = −5.669, *p* < 0.001**
Disagree	73	(48.3)	33	(21.9)	
Agree	69	(45.7)	112	(74.2)	
Strongly agree	3	(2.0)	5	(3.3)	
I am confident of managing patients with common mental health problems					
Strongly disagree	9	(6.0)	2	(1.3)	Z = −5.895, *p* < 0.001**
Disagree	95	(62.9)	52	(34.4)	
Agree	46	(30.5)	96	(63.6)	
Strongly agree	1	(0.7)	1	(0.7)	
I have enough time in my practice to handle patients with mental health problems					
Strongly disagree	14	(9.3)	12	(7.9)	Z = −0.952, *p* = 0.341
Disagree	89	(58.9)	86	(57.0)	
Agree	47	(31.1)	51	(33.8)	
Strongly agree	1	(0.7)	2	(1.3)	
My experiences regarding patients with mental health problems					
Generally positive	28	(18.8)	39	(26.0)	Z = −2.287, *p* = 0.022*
Neutral	109	(73.2)	106	(70.7)	
Generally negative	12	(8.1)	5	(3.3)	
I am confident of referring a patient with mental health problem to other health care professionals if necessary					
Strongly disagree	7	(4.6)	1	(0.7)	Z = −5.918, *p* < 0.001**
Disagree	65	(43.0)	28	(18.7)	
Agree	74	(49.0)	105	(70.0)	
Strongly agree	5	(3.3)	16	(10.7)	
Percentage of patients with mental health problems being recommended by me to western medicine doctors					
0 %	88	(71.5)	86	(74.1)	Z = −0.244, *p* = 0.807
1–10 %	21	(17.1)	18	(15.5)	
11–20 %	5	(4.1)	6	(5.2)	
21–30 %	2	(1.6)	2	(1.7)	
> 30 %	7	(5.7)	4	(3.5)	

*p < 0.05; **p < 0.001

Qualitative responses to the open-ended questions illustrated the causes of improvement, which included higher awareness of psychosomatic symptoms, better understanding of classification of mental disorders, and management approaches shared by western medicine primary care doctors and psychiatrists.

*Some small things could indicate a mental problem which I never knew before.*

*The contents of the course facilitate me in differentiating whether a patient suffers from mental health problem and how to manage it. I’m particularly inspired by the talks on OCD, bipolar disorder and schizophrenia.*

*The cases shared by the doctors. Most doctors share their experiences and skills in tackling the problems.*

Apart from applying the western medicine approach, some TCM practitioners realized their potential roles in providing care to mental health patients.

*Extraordinary; open-minded, and not having the western dominant attitude.*

*Diagnosis of mental health problems and the side effects which occur after taking [western medicine] pills. I realize we can give herbs or acupuncture to decrease [side effects] and make patients feel better.*

On the other hand, difficulties in understanding medical terms in English and indications of western medications were reported by some participants.

*Too many English terms of western medication and diagnosis which are not well understood.*

*The knowledge gap in western medications makes it difficult to actually understand what the drugs are for.*

The proportion of participants being confident of referring a patient with mental health problem to other health care professionals increased (52.3 % vs 80.7 %). However, there was no significant change in percentage of mental health patients being recommended by the participants to western medicine doctors due to the lack of a formal referral system in Hong Kong. Besides, no significant change was indicated in terms of time sufficiency in handling patients with mental health problems. These barriers are explained in the qualitative responses.

*There is no system for TCM practitioners to formally refer patients to western medicine doctors.*

*No clue on what should be done if the patient can’t be helped*

*Insufficient time to take a full history of patients with mental health problems*

*Clinical work is too busy for me to understand the background of each patient*

Besides, negative attitude of some patients, which could be due to their mental illness, might discourage the TCM practitioners from caring their mental health problems.

*The negative/rude attitude of the patient is so discouraging that the doctor would lose their patience to communicate with the patient*

Suggestions were made by the participants to further enhance the learning outcomes of the course. They included delivering a more comprehensive session on cognitive-behavioral therapy and counselling, having more pragmatic approach with emphasis on integrating TCM with western medicine in helping patients with mental disorders, and involving TCM practitioners who are experienced in handling mental health problems as tutors.

### Relationship between participants’ background characteristics and reported learning outcomes

Participants were classified into two groups according to their reported learning outcomes (Table 2). A total of 66.9 % of participants marked some increase in confidence in recognizing patients with psychological problems, diagnosis or/and management of patients with common mental health problems. Participants who had received their undergraduate TCM training in Hong Kong (*p* = 0.0085), with no previous postgraduate qualification (*p* = 0.0120), or felt comfortable to be taught by western medicine doctors (*p* = 0.0215) were significantly more likely to show improvement.

**Table 2:** Association between respondents’ background characteristics and reported learning outcomes

	Increased confidence in recognition, diagnosis or/and management				Fisher’s exact test (Yes vs No) *p*-value
	Yes (*n* = 101)		No (*n* = 50)		
	N	(%)	N	(%)	
Personal background					
Gender					0.2955
Male	40	39.6	24	49.0	
Female	61	60.4	25	51.0	
Undergraduate TCM training					0.0085*
Hong Kong	93	92.1	36	75.0	
Mainland China	8	7.9	12	25.0	
Having postgraduate qualification					0.0120*
Yes	26	27.4	21	50.0	
No	69	72.6	21	50.0	
Reasons for studying this Course					
Meet job demand					0.3753
Least important	3	3.0 %	2	4.0 %	
Unimportant	33	32.7 %	10	20.0 %	
Important	55	54.5 %	31	62.0 %	
Very important	10	9.9 %	7	14.0 %	
Interest in community psychological medicine					0.4207
Least important	1	1.0 %	1	2.0 %	
Unimportant	1	1.0 %	2	4.0 %	
Important	52	51.5 %	27	54.0 %	
Very important	47	46.5 %	20	40.0 %	
Interest in western medicine					0.6594
Least important	1	1.0 %	1	2.0 %	
Unimportant	19	19.0 %	8	16.0 %	
Important	57	57.0 %	26	52.0 %	
Very important	23	23.0 %	15	30.0 %	
Attitudes towards western medicine					
I am comfortable to be taught by western medicine doctors					0.0215*
Strongly disagree	1	1.0 %	0	0.0 %	
Disagree	3	3.0 %	8	16.0 %	
Agree	66	65.3 %	31	62.0 %	
Strongly agree	31	30.7 %	11	22.0 %	
Postgraduate courses taught by western medicine doctors can help me look after my patients with common mental health problems					0.1029
Strongly disagree	3	3.0 %	1	2.0 %	
Disagree	2	2.0 %	4	8.0 %	
Agree	76	76.0 %	41	82.0 %	
Strongly agree	19	19.0 %	4	8.0 %	
I am confident of integrating western medicine approach in my daily clinical practice of TCM					0.9222
Strongly disagree	2	2.0 %	1	2.0 %	
Disagree	18	17.8 %	7	14.0 %	
Agree	69	68.3 %	35	70.0 %	
Strongly agree	12	11.9 %	7	14.0 %	
Views on providing mental health care					
I have enough time in my practice to handle patients with mental health problems					0.4458
Strongly disagree	11	10.9 %	3	6.0 %	
Disagree	58	57.4 %	31	62.0 %	
Agree	32	31.7 %	15	30.0 %	
Strongly agree	0	0.0 %	1	2.0 %	
My experiences regarding patients with mental health problems are					0.5142
Positive	16	16.2 %	12	24.0 %	
Neutral	74	74.7 %	35	70.0 %	
Negative	9	9.1 %	3	6.0 %	

*p < 0.05

## Discussion

This study on a 5-year sample showed that 66.9 % of the participants perceived their mental health care management had improved after attending the 10-session training course. The results reinforced the positive outcomes shown by our previous study [23]. Besides, the influencing background characteristics, enablers and barriers of improvement were identified.

Behavioral studies had illustrated the positive association between learning outcomes and flexibility in learning [24]. Mills et al. [25] pointed out the importance of intellectual openness for TCM practitioners to overcome the discrepancies in the practice of TCM and western medicine. In our study, participants who were more comfortable learning from western medicine doctors perceived greater gains from the programme. The medium of instruction, which was a mixture of Cantonese and English, could have an impact as well. It could be more suitable for practitioners who graduated from local institutions in Hong Kong. Previous research found that whether the teaching delivered was tailor-made to the practitioners was crucial for the learning outcomes [26]. It was therefore more likely for our students to have enhanced confidence and better integration into their clinical practices when a mixture of Cantonese and English was used for the teaching.

The reasons of improvement were described in the qualitative responses. The open-minded attitudes of the teachers and case sharing encouraged the TCM practitioners to learn and apply western medicine approach. Some TCM practitioners also realized the possibility in enhancing care to the mental health patients by TCM approach. On the other hand, barriers were mentioned by some participants. Apart from learning factors such as difficulties in understanding medical terms in English and the knowledge gap between Chinese and Western medicine, some encountered problems of consultation time constraints and patients’ negative attitude. In fact, these are typical barriers encountered by western medicine primary care doctors too [27]. However, significant improvement in time efficiency (55.8 % before the course vs 72.1 % after) had been reported in our study on mental health course for western medicine doctors [5]. In the future, the teachers of the course should address these practical barriers and share their personal experience with the TCM practitioners to explore possible solutions. Some participants preferred to have a more comprehensive session on cognitive-behavioral therapy and counseling. While this might be of benefits to some students, such a session would be beyond the learning objectives of this 10-session training course which aimed to provide a basic training in handling mental health patients. The most fundamental objective was to enable TCM practitioners to recognize psychological problems and psychosomatic symptoms, and not to mistreat them as purely physical illnesses. We, however, expected some students would be motivated to pursue further training in specific areas. This study also found that the students’ increased intention in referring mental health patients did not lead to a significant increase in referral rate. The atmosphere in the real clinical setting was less optimal than that of the classroom where the teachers generally supported integration of TCM and western medicine. The lack of a formal referral system, as well as the western medicine doctors’ concerns about applying TCM when the patients are taking western medications [28], would discourage the collaboration. As suggested by some students, the course can involve TCM practitioners as tutors. This may help to provide the students and course organizers the strategies to overcome the barriers of collaboration.

This study has several limitations. First, due to the lack of a control group, the specific effects of the training course could not be quantified fully. Second, the findings were based on self-reported data from the TCM practitioners. Other measurements like patient questionnaires, diagnostic screening tools [29], and skill assessment using standardized role-played scenarios [7] would be helpful to further confirm the results of this study. Third, despite the larger sample size, our sampled group was the new and major upcoming generation of TCM practitioners in Hong Kong from the public health care setting.

## Conclusion

The Course has positive impact on TCM practitioners in handling mental health patients. The main areas of improvement included increased awareness of mental symptoms, better understanding of classification of mental disorders and management approaches. On the other hand, barriers included difficulties in understanding medical terms in English, consultation time constraints, and a lack of formal referral system to psychiatrists. The findings are useful for designing similar trainings on CAM practitioners in other countries.

## Additional files

Additional file 1: The course curriculum. (DOCX 14 kb)
Additional file 2: Questions of pre-course and post course surveys. (DOC 143 kb)

## Abbreviations

CAM: Complementary and alternative medicine
TCM: Traditional Chinese Medicine
