# Supplementary material for: Five-year outcomes of western mental health training for Traditional Chinese Medicine practitioners
Source: BMC Psychiatry. 2016 Oct 26;16:363. doi: 10.1186/s12888-016-1080-6 (PMC5081669; doi:10.1186/s12888-016-1080-6)
Supplement: Additional file 1: — The course curriculum. (DOCX 14 kb) [file 12888_2016_1080_MOESM1_ESM.docx]

**The Course Curriculum**

| **Course length** | Ten weekday afternoons within three months  Two hours per session |
| --- | --- |
| **Objectives** | Designed to enrich students with theoretical and updated knowledge of common psychological problems with specific reference to their diagnosis, basic management and referral |
| **Content** | Ten seminars on topics related to common psychological problems and psychotherapy  Overview and interview skills  Stress related disorders  Mood disorders including bipolar disorders  Somatoform disorders  Panic and phobic disorders  Obsessive-compulsive and related disorders  Psychotherapy  Substance abuse including alcoholism  Psychotic disorders  Sleep disorders |
