# Supplementary material for: Five-year outcomes of western mental health training for Traditional Chinese Medicine practitioners
Source: BMC Psychiatry. 2016 Oct 26;16:363. doi: 10.1186/s12888-016-1080-6 (PMC5081669; doi:10.1186/s12888-016-1080-6)
Supplement: Additional file 2: — Questions of pre-course and post course surveys. (DOC 143 kb) [file 12888_2016_1080_MOESM2_ESM.doc]

# Questions of pre-course and post-course surveys

# *Pre-course survey questions*

[questionnaire code]

| *Please tick the most appropriate box for the following items.*  **Clinical practice characteristics** | |  |
| --- | --- | --- |
|  |  |  |
| 1. | I am confident of recognizing patients with psychological problems |  |
|  | Strongly disagree | □ |
|  | Disagree | □ |
|  | Agree | □ |
|  | Strongly agree | □ |
|  |  |  |
| 2. | I am confident of ***diagnosing*** patients with common mental health problems (e.g. anxiety, depression, sleeping disorder) |  |
|  | Strongly disagree | □ |
|  | Disagree | □ |
|  | Agree | □ |
|  | Strongly agree | □ |
|  |  |  |
| 3. | I am confident of ***managing*** patients with common mental health problems |  |
|  | Strongly disagree | □ |
|  | Disagree | □ |
|  | Agree | □ |
|  | Strongly agree | □ |
| 4. | I am confident of referring a patient with mental health problem to other healthcare professionals if necessary |  |
|  | Strongly disagree | □ |
|  | Disagree | □ |
|  | Agree | □ |
|  | Strongly agree | □ |
|  |  |  |
| 5. | Percentage of my patients with mental health problems being recommended by me to western doctors | ____% |
|  |  |  |
| 6. | I have enough time in my practice to handle patients with mental health problems |  |
|  | Strongly disagree | □ |
|  | Disagree | □ |
|  | Agree | □ |
|  | Strongly agree | □ |
|  |  |  |
| 7. | I am comfortable to be taught by western doctors |  |
|  | Strongly disagree | □ |
|  | Disagree | □ |
|  | Agree | □ |
|  | Strongly agree | □ |
|  |  |  |
| 8. | Postgraduate courses taught by western doctors can help me look after my patients with common mental health problems |  |
|  | Strongly disagree | □ |
|  | Disagree | □ |
|  | Agree | □ |
|  | Strongly agree | □ |
|  |  |  |
| 9. | I am confident of integrating western medicine approach in my daily clinical practice of TCM |  |
|  | Strongly disagree | □ |
|  | Disagree | □ |
|  | Agree | □ |
|  | Strongly agree | □ |
|  |  |  |
| 10. | My experiences regarding patients with mental health problems are |  |
|  | Generally positive | □ |
|  | Neutral | □ |
|  | Generally negative | □ |
|  |  |  |

| **Reasons for studying this Course** | | *least important* | *unimportant* | *important* | *very important* |
| --- | --- | --- | --- | --- | --- |
|  |  | 1 | 2 | 3 | 4 |
| 1. | Meet job demand | □ | □ | □ | □ |
| 2. | Interest in community psychological medicine | □ | □ | □ | □ |
| 3. | Interest in western medicine | □ | □ | □ | □ |

| **Please provide some information about yourself which will remain strictly anonymous** | | |
| --- | --- | --- |
| 1. | Year of graduation from medical school | ⬜⬜⬜⬜ *(yyyy)* |
| 2. | Gender | ⬜ Male ⬜ Female |
| 3. | Undergraduate TCM training was in | ⬜ Hong Kong ⬜ Mainland |
| 4. | Any postgraduate qualification  (please specify: _______________________________) | ⬜ Yes ⬜ No |

***Post-course survey questions***

[questionnaire code]

| *Please tick the most appropriate box for the following items.*  **Clinical practice characteristics** | |  |
| --- | --- | --- |
|  |  |  |
| 1. | I am confident of recognizing patients with psychological problems |  |
|  | Strongly disagree | □ |
|  | Disagree | □ |
|  | Agree | □ |
|  | Strongly agree | □ |
|  |  |  |
| 2. | I am confident of ***diagnosing*** patients with common mental health problems (e.g. anxiety, depression, sleeping disorder) |  |
|  | Strongly disagree | □ |
|  | Disagree | □ |
|  | Agree | □ |
|  | Strongly agree | □ |
|  |  |  |
| 3. | I am confident of ***managing*** patients with common mental health problems |  |
|  | Strongly disagree | □ |
|  | Disagree | □ |
|  | Agree | □ |
|  | Strongly agree | □ |
| 4. | I am confident of referring a patient with mental health problem to other healthcare professionals if necessary |  |
|  | Strongly disagree | □ |
|  | Disagree | □ |
|  | Agree | □ |
|  | Strongly agree | □ |
|  |  |  |
| 5. | Percentage of my patients with mental health problems being recommended by me to western doctors | ____% |
|  |  |  |
| 6. | I have enough time in my practice to handle patients with mental health problems |  |
|  | Strongly disagree | □ |
|  | Disagree | □ |
|  | Agree | □ |
|  | Strongly agree | □ |
|  |  |  |
| 7. | I am comfortable to be taught by western doctors |  |
|  | Strongly disagree | □ |
|  | Disagree | □ |
|  | Agree | □ |
|  | Strongly agree | □ |
|  |  |  |
|  |  |  |
| 8. | Postgraduate courses taught by western doctors can help me look after my patients with common mental health problems |  |
|  | Strongly disagree | □ |
|  | Disagree | □ |
|  | Agree | □ |
|  | Strongly agree | □ |
|  |  |  |
| 9. | I am confident of integrating western medicine approach in my daily clinical practice of TCM |  |
|  | Strongly disagree | □ |
|  | Disagree | □ |
|  | Agree | □ |
|  | Strongly agree | □ |
|  |  |  |
| 10. | My experiences regarding patients with mental health problems are |  |
|  | Generally positive | □ |
|  | Neutral | □ |
|  | Generally negative | □ |

**Open-ended questions**

1. What is the most important impact of the course on you?

____________________________________________________________________________

____________________________________________________________________________

____________________________________________________________________________

2. Have you experienced any challenges or barriers to implement what you learned in this course?

____________________________________________________________________________

____________________________________________________________________________

____________________________________________________________________________

3. Do you have any suggestions or comment for the course (e.g. topics, way of delivery)?

____________________________________________________________________________

____________________________________________________________________________

____________________________________________________________________________

***Please provide some information about yourself which will remain strictly anonymous***

| 1. | Year of graduation from medical school | ⬜⬜⬜⬜ *(yyyy)* |
| --- | --- | --- |
| 2. | Gender | ⬜ Male ⬜ Female |
| 3. | Undergraduate TCM training was in | ⬜ Hong Kong ⬜ Mainland |
| 4. | Any postgraduate qualification  (please specify: _______________________________) | ⬜ Yes ⬜ No |
